# Carotid Stiffness and Physical Activity in Elderly—A Short Report of the SAPALDIA 3 Cohort Study

**DOI:** 10.1371/journal.pone.0128991

**Published:** 2015-06-02

**Authors:** Seraina Caviezel, Julia Dratva, Emmanuel Schaffner, Christian Schindler, Simon Endes, Christine S. Autenrieth, Miriam Wanner, Brian Martin, Eric de Groot, Jean-Michel Gaspoz, Nino Künzli, Nicole Probst-Hensch, Arno Schmidt-Trucksäss

**Affiliations:** 1 Department of Sport, Exercise and Health, Div. Sports and Exercise Medicine, University of Basel, Basel, Switzerland; 2 Swiss Tropical and Public Health Institute, Basel, Switzerland; 3 University of Basel, Basel, Switzerland; 4 Institute of Social and Preventive Medicine, Physical Activity and Health Unit, University of Zurich, Zurich, Switzerland; 5 Imagelabonline, Science Centre, Amsterdam, The Netherlands; 6 Department of Community Medicine and Primary Care, University Hospitals of Geneva, Geneva, Switzerland; Universidad Pablo de Olavide, Centro Andaluz de Biología del Desarrollo-CSIC, SPAIN

## Abstract

**Introduction:**

Regular physical activity has been shown to reduce cardiovascular disease risk in the general population. While smaller studies in specified groups (highly trained versus untrained individuals) indicate a certain dose-dependent effect of physical activity on the reduction of carotid stiffness (an indicator of subclinical vascular disease), it is unclear whether this association is present in a representative sample. Thus, we investigated this question cross-sectionally in participants from the population-based Swiss Cohort Study on Air Pollution And Lung and Heart Diseases In Adults (SAPALDIA).

**Methods:**

Self-reported total, moderate and vigorous physical activity and distensibility as a measure of local arterial stiffness among 1636 participants aged 50 to 81 years without clinically manifest diseases were evaluated. Mixed regression models were used to examine associations of physical activity intensity with distensibility.

**Results:**

Vigorous physical activity, but not total nor moderate physical activity, was significantly associated with increased distensibility (= reduced carotid stiffness) in univariate analyses (percent change in the geometric mean and 95% confidence interval per 1 standard deviation increment in vigorous physical activity = 2.54 (0.69; 4.43), p<0.01; in total physical activity = 1.62 (-0.22; 3.50), p = 0.08; in moderate physical activity = 0.70 (-1.12; 2.56), p = 0.45). These associations disappeared when we additionally adjusted for age.

**Conclusion:**

After adjustment for the most important confounders and risk factors, we found no evidence for an association of physical activity with carotid stiffness in the general middle aged to elderly population.

## Introduction

Physical inactivity is a generally accepted cardiovascular risk factor for cardiovascular disease (CVD), which causes a high percentage of global mortality [1–3]. CVD begins with structural and functional changes of the arterial system commonly known as the atherosclerotic process [4,5]. The vascular damage accumulated over time can be assessed as arterial stiffness, which can be directly measured in the common carotid artery [6].

While regular physical activity (PA) has been shown to reduce CVD risk in the general population, results of inverse associations between an increased PA level and a reduced carotid stiffness were mostly limited to two or three healthy groups with specific group characteristics such as highly trained versus untrained subjects [7–14]. Although these reports suggest a certain dose-dependent effect of PA on carotid stiffness [7–14], the specific group distinctions constrict a more generalizable conclusion.

Thus, the aim of this study was to assess whether an increasing amount of self-reported total, moderate and vigorous PA is associated with reduced carotid stiffness in a general middle-aged and elderly population without a diagnosis of CVD from the Swiss Cohort Study on Air Pollution And Lung and Heart Diseases In Adults (SAPALDIA).

## Material and Methods

The SAPALDIA cohort study started in 1991 and is an ongoing Swiss multicentre cohort with a population-based random sample of adults [15,16]. This short report is based on the second follow-up assessment (SAPALDIA 3), which was conducted in 2010–2011.

Bilateral ultrasound B-mode scans of the common carotid artery were assessed in 3489 participants aged between 50–81 years at the time of examination. Centrally trained and certified sonographers used the same standardised ultrasound instruments (UF-870 machine LA385-16 MHz array transducer, Fukuda Denshi, Japan) and scan protocols. Ultrasound scans were analysed offline by expert readers evaluating carotid structure in a standardized 1cm segment across at least one heart cycle [17,18]. Detailed information about the examination and evaluation process are described elsewhere [17,18].

Distensibility was calculated as ([1/kPa] = ((2 × deltaLD × dLD)+(deltaLD)^2^)/(PP× dLD^2^)) [19], where deltaLD is the systolic-diastolic lumen diameter difference, dLD the diastolic lumen diameter and PP the pulse pressure (measured immediately after ultrasound examination, OMRON 705IT, OMRON HEALTHCARE, Kyoto, Japan). Lower values of distensibility correspond to an increased carotid stiffness. Good reproducibility of distensibility measurement was shown in 165 SAPALDIA 3 participants (coefficient of variation 12.14%; intraclass correlation coefficient 0.77) [18].

**Fig 1:**
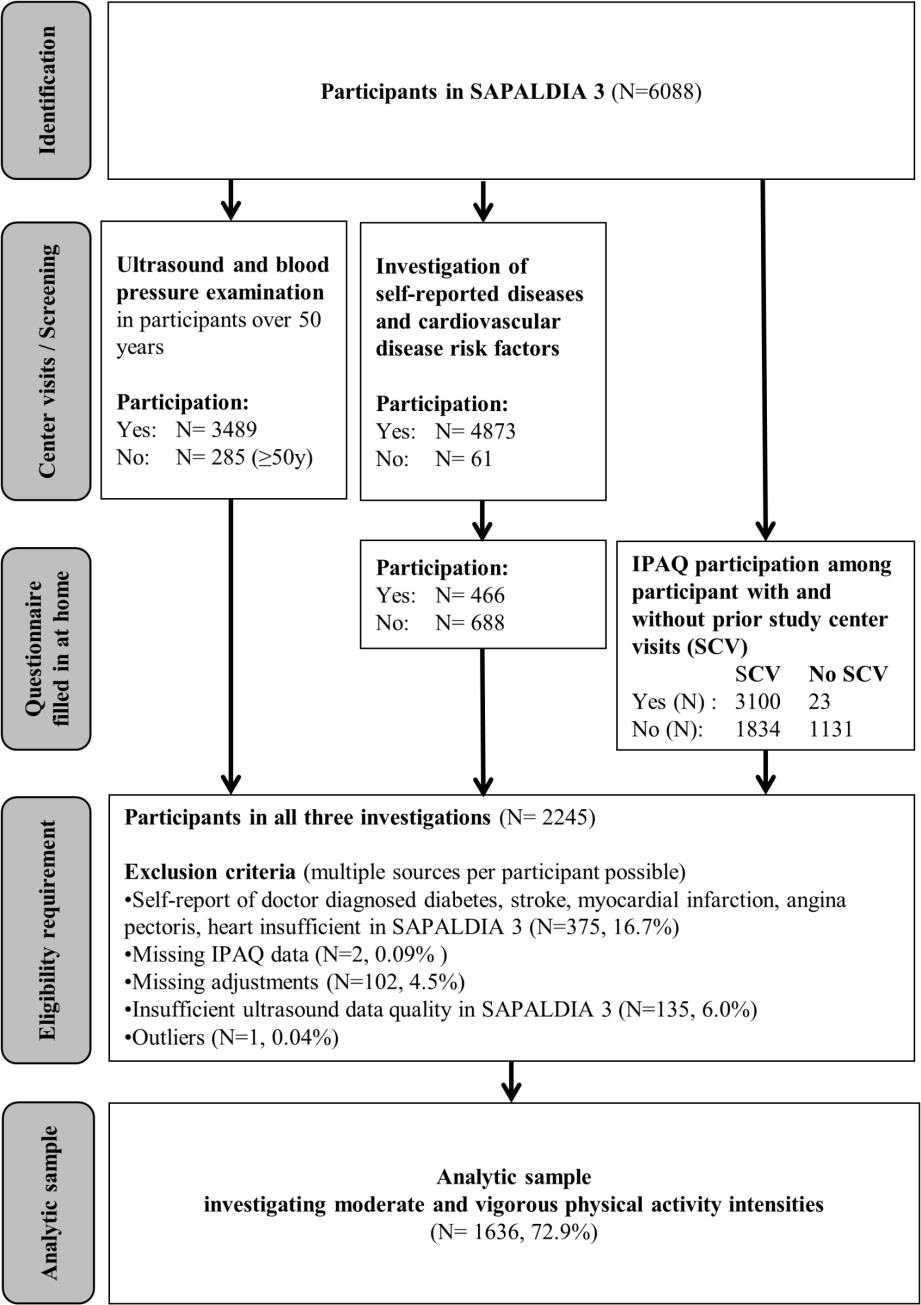
Flow chart of subject inclusion. SCV = prior study center visits, IPAQ = International Physical Activity Questionnaire, PA = physical activity, affected adjustments = body mass index, mean arterial pressure, heart rate, smoking.

PA parameters were assessed using the validated long form of the International Physical Activity Questionnaire (IPAQ) [20,21]. Moderate and vigorous PA values of at least 10 minute bouts were derived from 27 items and four domains (work, transport, domestic/garden, leisure time) [22]. Values above 1260 min/week (3hours/day) were truncated [22]. The sum of moderate and vigorous PA intensity was calculated and defined as total PA. Participants were asked to fill in the IPAQ at home. Fig 1 shows that the participation rate was much lower when questionnaires were filled in at home compared to those participants who previously visited the study centers. The study complies with the declaration of Helsinki and ethical clearance was obtained and approved from the respective cantonal ethical committees (Aargau, Basel, Geneva, Grisons, Ticino, Valais, Vaud and Zurich) and participants gave written informed consent according to their preferences either globally for all examinations or separately for single assessments.

### Statistical analysis

Data from 1636 participants were available for analyses after exclusion of individuals with doctor diagnosed diabetes, stroke, myocardial infarction, angina pectoris or heart failure at time of ultrasound examination or missing ultrasound, IPAQ or additional covariate data (Fig 1). Distensibility was skewed on the original scale and was normalised by log-transformation. Statistical significance was defined for p-values <0.05.

The associations of distensibility with PA parameters were examined using multiple mixed linear regression models with standardised covariates. Model 1 was an univariate model of distensibility and PA determinants; model 2 additionally adjusted for sex and age; model 3 included further adjustments for cardiovascular risk factors (heart rate, body mass index (BMI), mean arterial blood pressure and smoking), medication (yes/no: antihypertensive, lipid lowering, and kidney disease treatment) and SAPALDIA study centres (as random intercept to account for regional clustering). We repeated the main analyses considering other carotid stiffness parameters, namely beta stiffness index, Peterson’s elastic modulus and Young’s elastic modulus. However, conclusions did not differ from distensibility and thus, only results of distensibility are presented in this short report.

All analyses were performed using the statistical software STATA (StataCorp, Release 12. Statistical Software, College Station, TX: StataCorp LP, Texas, USA). The analytical data set and the statistical code are available from the corresponding author upon request, since ethics approval and participants consent does not allow public sharing of data (http://www.sapaldia.ch).

## Results

**Fig 2:**
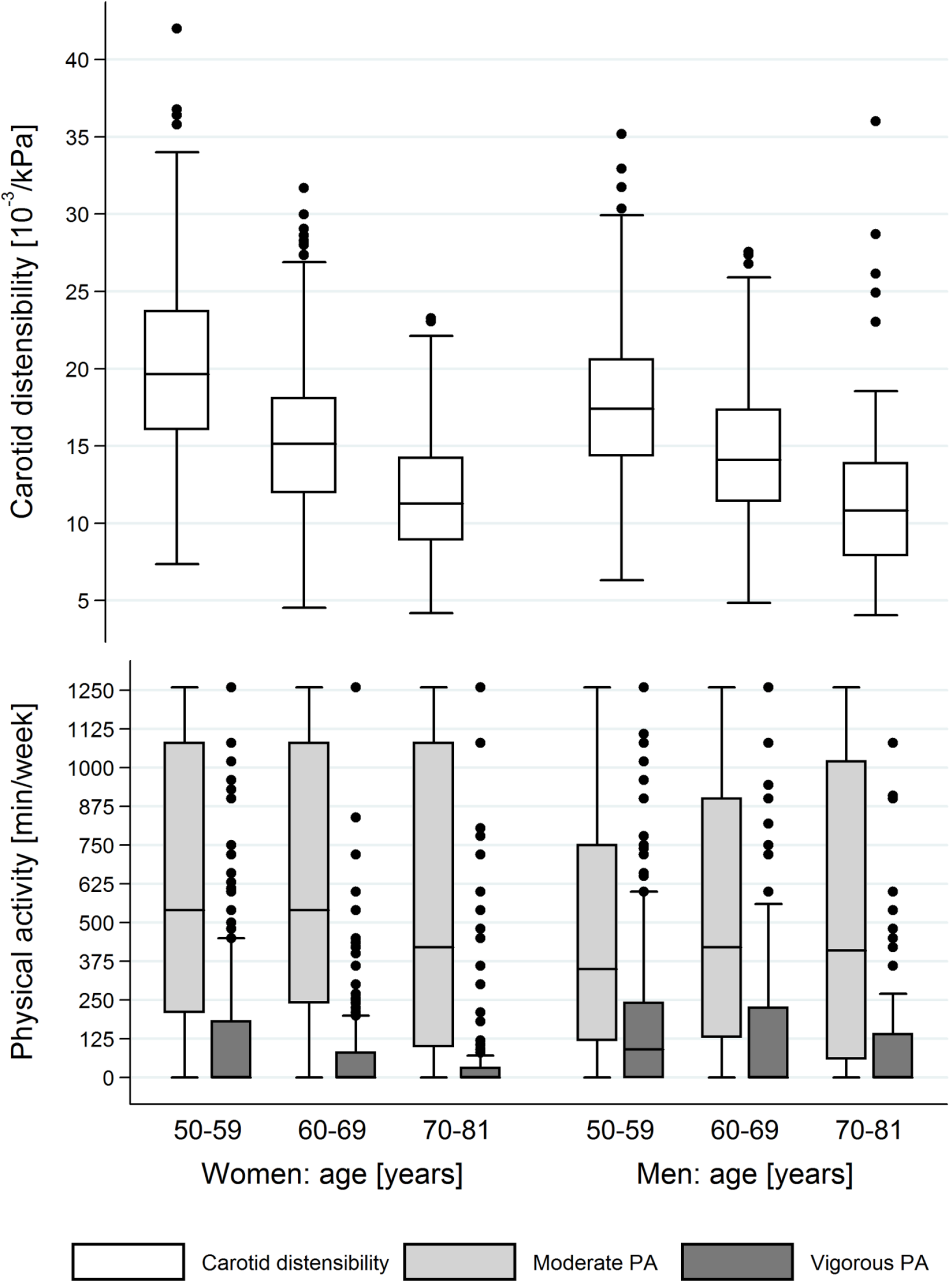
Box plots of distensibility and physical activity (PA) for both sexes across three 10-year age categories.

Characteristics of study participants for males and females are shown in Table 1. Box plots of distensibility values and moderate and vigorous PA for both sexes and across three 10-year age categories are presented in Fig 2. Compared to the youngest age group, distensibility and vigorous PA were lower in older age groups for both sexes (p<0.05). Moderate PA did not show an age-associated decline. Within each age group, vigorous PA was significantly lower in women than in men (p<0.05). No differences for distensibility and moderate PA values were found in the oldest age group for both sexes (p>0.1). In the youngest and middle-aged groups, women had on average a higher distensibility and moderate PA compared to men (p<0.05).

**Table 1:** Characteristics of the study population by sex.

Characteristics	Units	Men	Women
**N participants**	N (%)	712 (43.5)	924 (56.5)
**No medication intake**	N (%)	463 (39.9)	696 (60.1)
**Medication intake**	N (%)	249 (52.2)	228 (47.8)
**Age**	Mean (SD) years	63.0 (7.6)	63.0 (7.8)
**Height**	Mean (SD) cm	175.1 (6.3)	162.0 (6.3)
**Weight**	Mean (SD) kg	81.8 (11.9)	65.7 (12.3)
**Body mass index**	Mean (SD) kg/m2	26.7 (3.5)	25.1 (4.6)
**Diastolic BP**	Mean (SD) mmHg	80.1 (9.4)	76.5 (9.1)
**Systolic BP**	Mean (SD) mmHg	137.5 (17.3)	131.5 (17.9)
**Mean BP**	Mean (SD) mmHg	99.2 (11.2)	94.8 (11.1)
**Pulse pressure**	Mean (SD) mmHg	57.4 (12.1)	55.1 (13.1)
**Heart rate**	Mean (SD) bpm	68.1 (10.6)	69.1 (9.2)
**Smoking**	Median (p25, p75) pack years	2.5 (0, 24)	0 (0, 12)
**Distensibility**	Median (p25, p75) 10^-3^/kPa	14.5 (11.4, 18.1)	15.8 (12.0, 20.1)
**Total PA**	Median (p25, p75) min/week	510 (180, 1105)	590 (240, 1195)
**Moderate PA**	Median (p25, p75) min/week	360 (120, 00)	510 (193, 1080)
**Vigorous PA**	Median (p25, p75) min/week	25 (0, 225)	0 (0, 113)

Numbers (N), mean value and standard deviation (SD), median and interquartile range (p25, p75), blood pressure (BP), physical activity (PA).

Detailed estimates of the associations between PA intensities and distensibility from different mixed linear models (models 1–3) are depicted in Table 2. We found significant univariate associations (model 1) between vigorous PA and distensibility, but not for total PA and moderate PA (percent change in the geometric mean per 1 standard deviation increment increase in vigorous PA = 2.54, p<0.01; in total PA = 1.62, p = 0.08; in moderate PA = 0.70, p = 0.45). In all mixed linear regression analyses, associations of different PA determinants with distensibility disappeared when age was included in the model (Table 2, model 2 and 3).

**Table 2:** Standardized estimates of associations between different physical activity (PA) determinants and distensibility adjusted for different covariates.

Outcome: distensibility	Moderate PA		Vigorous PA		Total PA	
	CGM (95% CI) [%]	p-value	CGM (95% CI) [%]	p-value	CGM (95% CI) [%]	p-value
Model 1	0.70 (-1.12; 2.56)	0.45	2.54 (0.69; 4.43)	<0.01	1.62 (-0.22; 3.50)	0.08
Model 2	0.17 (-1.34; 1.70)	0.83	-0.24 (-1.76; 1.31)	0.76	0.04 (-1.46; 1.57)	0.96
Model 3	0.40 (-0.88; 1.70)	0.54	-0.52 (-1.80; 0.78)	0.43	0.12 (-1.16; 1.41)	0.86

Results are expressed as percentage change in the geometric mean (CGM) per 1 standard deviation increment with 95% confidence interval (CI); model 1 = univariate association between distensibility and moderate PA, vigorous PA and total PA; model 2 = adjusted for age and sex; model 3 = additional adjustments for medication intake, mean arterial blood pressure, body mass index, heart rate, smoking and a random effect for study centers to account for clustering.

## Discussion

We observed no association of self-reported PA with distensibility in individuals aged 50 to 81 years of the SAPALDIA cohort free of CVD diagnosis. Only vigorous PA was significantly associated with reduced carotid stiffness in univariate analyses. However, these associations disappeared after adjustment for age.

Our results are principally in line with a report of ‘The Atherosclerosis Risk in Community’ (ARIC) cohort study which was also cross-sectional in design [14]. The authors found no association of PA with carotid stiffness in the overall population. Only in a sub-analysis, vigorous PA was weakly associated with reduced carotid stiffness independent of risk factors and age [14]. In contrast, far smaller cross-sectional studies seemed to show much stronger inverse associations of increased PA with reduced carotid stiffness parameters [8–14,23]. In addition, these results suggest a certain dose-dependent reduction of carotid stiffness with more vigorous PA even at older age [11,13]. However, these findings were primarily obtained by comparing extremes of PA (trained middle-aged to older athletes vs. sedentary peers) [9–11,13], and the fact that some of the very strong effects were found in small studies raises concern about publication bias. Except for ARIC, these small studies with highly selected samples probably do not reflect the general population, limiting the transfer of their results from athletes to normal individuals.

Positive effects of PA on cardiovascular risk factors and CVD were already widely investigated [3,24–28] and different plausible associations of cardiovascular risk factors and carotid stiffness were shown by our group in the same study population [29]. We were convinced to find a clear independent positive contribution of PA on carotid stiffness due to favourable mechanisms via the nitric oxide pathway [30–33], and conserving effects on elastin and collagen content against adverse wall remodelling [33,34]. In contrast to previous findings and to our a priori defined hypothesis, we could not show any effect of PA on arterial stiffness.

### Strengths and limitations

Results of this short report are based on a large sample size using highly standardised ultrasound examination procedures to assess carotid stiffness. Besides the fact that IPAQ is already a validated tool for population wide PA assessment [20,21], a separate IPAQ validation within the SAPALDIA 3 setting including elderly individuals was performed and results confirmed the previous findings (Wanner et al. submitted). However, self-reported PA over the last seven days might lead to bias since PA intensity is based on subjective assessment. People might consider themselves active based on their life-long activity level, which may have changed with age. Overall, the degree of misclassification might be strong enough to prevent small effects of PA from being detected. However, measurement of PA in a large sample is challenging and costly and a validated questionnaire is a highly feasible solution to assess PA in a cohort. In line with other larger studies investigating PA and carotid stiffness, our study was limited by its cross-sectional design and this does not solve the challenge of possible reverse causality. Even though we excluded participants with known pre-existing CVD, there is still the chance that an unknown unfavourable health status affects the association between PA and carotid stiffness. Thus, to address reverse causality, we suggest to assess PA longitudinally before measuring carotid stiffness.

## Conclusion and Perspectives

To our knowledge, this is the second epidemiological study investigating the associations of PA, risk factors and age on carotid stiffness assessed in an aging population without diagnosed CVD. Although we hypothesized an independent beneficial effect of PA on arterial stiffness in the common carotid artery, only an increasing amount of vigorous PA (and not total or moderate PA) was associated with reduced carotid stiffness and the association was no longer present after adjusting for age. Since only small intervention studies in specific populations showed a beneficial effect of PA on carotid stiffness, we recommend to investigate PA determinants in correlation with carotid stiffness longitudinally including a representative study sample.
